# Exploring older adults’ perceptions of intergenerational relationships and programs: a qualitative comparative analysis of participants and non-IGP participants

**DOI:** 10.3389/fpubh.2025.1733311

**Published:** 2026-01-19

**Authors:** Jasmine Yee Ru Cheng, Elizabeth Skylar Loo, Audrey Shu Ting Kwan, Wan Qi Yee, Zheng Kwok Sow, Joanne Sze Win Lee, Jamaica Pei Ying Tan, Nigel Teo, Kennedy Yao Yi Ng, Lian Leng Low

**Affiliations:** 1 Division of Population Health and Integrated Care, Singapore General Hospital, Singapore, Singapore; 2 Department of Physiotherapy, Singapore General Hospital, Singapore, Singapore; 3 Division of Medical Oncology, National Cancer Centre, Singapore, Singapore; 4 Oncology and Medicine Academic Clinical Programme, DUKE-NUS Medical School, Singapore, Singapore; 5 Department of Global Health and Population, Harvard T.H Chan School of Public Health, Boston, MA, United States; 6 TriGen, Singapore, Singapore; 7 Family Medicine Academic Clinical Program, Duke-NUS Medical School, Singapore, Singapore; 8 Research and Translation Innovation Office, SingHealth Community Hospital, Singapore, Singapore

**Keywords:** generation gap, intergenerational programs, intergenerational relationships, motivation, older adults

## Abstract

**Introduction:**

As the global population ages at an unprecedented rate, rapid shifts in demographics and evolving family structures have placed increasing pressure on countries to ensure the health and well-being of an aging population. Intergenerational programs have emerged as a potential solution in improving the physical, social, and psychological outcomes of older adults. While research has primarily focused on intergenerational relationships within care facilities, familial context, or participants of intergenerational programs, few studies have examined these relationships in broader community settings. This study aimed to understand and compare the experiences, facilitators, barriers, and perceptions of older adults who have participated in intergenerational programs before with those who have not.

**Methods:**

A qualitative descriptive approach with semi-structured interviews was conducted with 15 older adults who participated in intergenerational programs and 10 older adults who did not participate in intergenerational programs via purposive sampling. Data were analyzed thematically using inductive coding.

**Results:**

The themes were organized into three themes: (1) perceptions of young adults and intergenerational relationships, (2) perception of generation gap and navigating intergenerational relationships, and (3) facilitators and barriers of intergenerational relationships and programs. A key finding was that participation in intergenerational programs fostered positive impressions of young adults, but older adults’ preference for casual interactions over deeper intergenerational relationships limited the sustainability and quality of the relationships.

**Conclusion:**

In conclusion, the study highlighted how older adults’ experiences and perceptions with intergenerational relationships and programs are shaped by both contact and motivation. Results indicated that contact alone may be insufficient, highlighting the need for a shift in mindset to view intergenerational relationships and programs as meaningful and rewarding.

## Introduction

The global population is aging at an unprecedented rate. By the late 2070s, the number of older adults (aged 65 years and older) is projected to reach 2.2 billion, surpassing the number of individuals under 18 years old (1). This demographic shift not only reflects declining fertility rates but also rising life expectancy and reduced mortality rates (2). Aging populations are often associated with higher health and social needs. Notably, older adults face several public health challenges. This includes a growing burden of chronic disease, an increase in mental and cognitive health issues, escalation of harmful ageist stereotypes and attitudes, and heightened vulnerability to social isolation, reducing older adults’ quality of life and well-being (2, 3).

In Singapore, 1 in 4 individuals is expected to be aged 65 years and older by 2030, with parallel age-related challenges (4). Singapore, shaped by Confucianism ideals, places strong emphasis on filial piety, cultural and moral norms, values, and practices centered around respect and providing care for one’s parents or elders (5, 6). Yet, rapid shifts in demographics and evolving family structures have placed increasing pressure on these traditional norms. As families become smaller and caregiving expectations shift, concerns about the health and well-being of an aging population have intensified (7). This is especially evident in Asian cultures where adult children are traditionally responsible for supporting aging parents. These emerging pressures call for community-based and behavioral interventions that reimagine caregiving beyond the family unit, strengthening relationships between generations that facilitate healthy aging in later life.

Intergenerational programs (IGPs) have emerged as a potential solution. IGPs, defined as a medium to facilitate intentional and continuous exchange of resources and learning between the younger and older generations, aim to strengthen intergenerational ties while addressing the health and social needs of older adults (8). Grounded in Allport’s Intergroup Contact Theory, these programs leverage exposure, increased frequency of contact, and growing familiarity to reduce negative attitudes and foster positive attitudinal change. In particular, this theory suggests that mere exposure, through increased frequency of contact and familiarity with members of the other group, enhances liking for them (9, 10). Evidence suggests that participation in IGPs reduces age stereotyping, creating cooperative contact that challenges deficit narratives about aging, and improves intergenerational solidarity through greater cross-age trust, reciprocity, and shared norms (11–13). Older adults also showed improved psychological outcomes, including reduced anxiety and an increase in a sense of self-worth (11, 12, 14) . IGPs have demonstrated efficacy in reducing feelings of social isolation and loneliness, which have shown to reduce older adults’ risk of heart diseases and high blood pressure (13, 16–19). Lastly, previous studies on HealthStart, a Singapore layperson-led trigenerational health coaching program for older adults, demonstrated that IGPs have the ability to facilitate intergenerational transfer of health knowledge, enabling mutual learning, empowering older adults to manage their health independently, and strengthen intergenerational relationships (20, 21).

While at least 90% of older adults are residing in communities, a large proportion of research has primarily focused on older adults in residential care facilities (12). While Allport’s Intergroup Contact Theory offers a useful lens for understanding how intergenerational relationships form and how IGPs catalyze them, there is a need to understand intergenerational relationships outside the familial sphere, where interactions are voluntary rather than role obligated. The Socioemotional Selectivity Theory (SST), which posits that humans have the unique ability to monitor the passage of time, adjust their perceived time horizon with increasing age, and recognize that time eventually runs out (22), may provide further insight into older adults’ perception of intergenerational relationships. As older adults prioritize emotionally rewarding, familiar networks, their motivation to invest in new intergenerational relationships may be selective and contingent on other factors. The SST provides a nuanced view on their motivation for intergenerational relationships and programs (23). Lastly, studies on IGPs have primarily focused on participants, with few studies examining the perceptions of those who have not participated in IGPs, who may face different motivations, barriers, and perceptions toward intergenerational relationships and programs.

As such, with IGPs gaining traction, we sought to understand not just their benefits, but also compare the experiences, barriers, and motivations between participants and non-participants. While we integrated Allport’s Intergroup Contact Theory and SST, we also took an inductive approach to understand participants’ lived experiences. This allowed for a more open but nuanced exploration of the perceptions of intergenerational relationships and programs. Thus, we aimed to understand and compare the experiences, facilitators, barriers, and views of older adults who have participated in IGPs with those who have not.

## Materials and methods

### Study design

We employed a qualitative descriptive study design with an interpretive approach to explore and compare the experiences and perceptions of older adults who participated in IGPs with older adults who had not participated in IGPs. This approach was utilized to capture participants’ firsthand experiences and preserve the findings’ applicability to real-world settings.

Our research question sought to understand and compare the experiences, facilitators, barriers, and views of older adults who have participated in IGPs before with those who have not.

### Study site

The study was led by Singapore General Hospital (SGH) Division of Population Health and Integrated Care (PHIC), which coordinates care-integration efforts in Singapore’s Southeast region (24). Participants were recruited from different social service agencies or centers in the Southeast region of Singapore that organized activities, such as IGPs, catering to older adults.

### Sampling and recruitment

Participants were recruited from social service agencies or centers in the Southeast region of Singapore that were regular partners who served older adults who either participated or did not participate in IGPs. They were recruited via purposive sampling to ensure representation of varied backgrounds and perspectives. A sample size of 25 participants falls within the range of achieving data saturation, allowing for thorough exploration of themes while maintaining the depth of analysis. Recruitment was conducted through a publicity poster distributed by community organizations. The inclusion criteria were community-dwelling older adults (aged 60 years and above) who either had taken part in one of the above IGPs involving both older adults and young adults (15 to 35 years old) within the last 3 years (IGP participants) or not taken part in any IGPs involving both age groups within the last 3 years (non-IGP participants). Older adults who resided in institutional settings were excluded.

Interested individuals signed up through an online form or by telephoning a study team member with the number provided on the publicity poster. After which, a semi-structured in-person interview was scheduled at the participant’s convenience and conducted in a quiet environment in either English, Mandarin, or Malay. Written consent was obtained once study information was provided to the participant, all questions were answered, and participants were assured they could skip questions or withdraw at any time. We continued the recruitment process until we determined that data saturation had been achieved, where no additional information arose from the interviews.

### Data collection

The topic guide was developed through a combination of reviewing relevant literature and empirical evidence, as well as drawing on the collective expertise and prior research experience of the study team, to ensure that it addressed key issues while remaining sensitive to the local context. A summary of the interview guide is presented in Table 1.

**Table 1:** Summary of interview guide.

Topic	Sample interview questions for IGP participants	Sample interview questions for non-IGP participants
Participating in IGPs	Can you tell me more about the IGP you attended?	What do you think of IGPs? Will you be interested in joining such programs? What made you decide against joining the program? Can you share with me some of your thoughts and feelings behind why you decided not to join?
Interactions with Young Adults	Can you tell me more about your experiences interacting with young adults through the program(s) you took part in? In your experience, was it easy or hard to interact with young adults? How do you think we can better encourage older adults, like yourself, to join IGPs? And what is the most important factor?	Can you share about your experience interacting with young adults? What do you think went well? What are some memorable moments from interacting with young adults?
Friendship with Young Adults	What is your experience in building friendships with young adults like?	What is your experience in forming friendships with young adults like? Do you think that there might be a difference in the lives of older adults like you, if they had more opportunities to interact with and befriend young adults? How so?
Generation Gap	Is the generation gap something you believe you have experienced with young adults? How do you think young adults view you?	
Suggestions for IGPs	If you come up with activities to bring older adults and young adults in the community together/to encourage friendships between older adults and young adults, what will this look like?	

Five interviewers (WQ, ZK, JT, JL, and NT) experienced in qualitative data collection methods conducted one-to-one semi-structured interviews with participants. Interviews were conducted via Zoom Web Conferencing (25) or in-person, with each interview lasting between 60 to 90 min. For each interview, one study team member was the main interviewer, and the second team member was a moderator who took key notes, observing the participants’ tone and expression, and prompting further questions. All interviews were audio-recorded, translated, and transcribed verbatim by either a student intern, a research team member, or a third-party transcription service. All transcripts were verified by a study team member to ensure verbatim accuracy and redact identifiable information. Non-English transcripts were handled using a professional third-party bilingual translation and transcription service. All data were de-identified and saved on a set of electronic devices accessible only by the research team.

### Data analysis

Two team members (JC, EL) coded the transcripts using an inductive approach. Thematic analysis was utilized, based on Braun and Clarke’s six-step framework, to analyze the transcripts (26). QSR NVivo 14 Software was used for data management and analysis.

Each transcript was independently coded by the two investigators and then cross-checked by both investigators to address any discrepancies. Codes were blended iteratively to form themes through discussion within the research team. Final themes were refined and titled to ensure they sufficiently represented the data findings. We strengthened the trustworthiness of our study through three strategies: (i) ensuring credibility by maintaining methodological transparency and alignment with our research question, (ii) ensuring confirmability by implementing double-coding and inclusive data representation, and (iii) ensuring transferability by relating our findings to the literature.

### Research team and reflexivity

All research team members were affiliated with the Division of PHIC at SGH. The team comprised public-health researchers who had prior experience with intergenerational programming but had no formal authority over participants. Before data collection, the team explained the interview process, ensured that participants were in a comfortable setting, and invited open, judgment-free sharing. The research team also encouraged participants to share their responses openly and honestly, assuring them that there would be no judgment.

To enhance reflexivity during data collection, interviewers engaged in pre- and post-interview debriefing to reflect on how our perspectives might influence questioning and interpretation. The semi-structured interview guides increased consistency, and several investigators cross-checked responses to highlight and consider diverse perspectives. Mindful of power dynamics, we affirmed that participants’ views would be received openly and critically assessed our own positionality to limit bias in collection, analysis, and reporting.

### Ethics statement

Ethics approval was granted by the SingHealth Centralized Institutional Review Board (Reference No. 2021/2773).

## Results

A total of 15 older adults who participated in IGPs and 10 older adults who had not participated in IGPs were recruited and interviewed. Interviews were conducted from January to July 2022. A majority of IGP participants were unable to recall the exact IGPs they took part in, and were only able to provide brief descriptions of the programs they took part in. The nature of these programs varied in duration and outcomes, with most IGPs having young adults taking on the role as facilitators and older adults as beneficiaries and participating in recreational activities (e.g., arts and craft, befriending, etc.). The sample characteristics of the participants are presented in Table 2.

**Table 2:** Summary of characteristics of participants.

IGP participants (*n* = 15)	
Sex	
Male	4 (26.7%)
Female	11 (73.3%)
Age	
Mean age	77
Age range	54–89
Race	
Chinese	11 (73.3%)
Malay	4 (26.7%)

Non-IGP participants (*n* = 10)	
Sex	
Male	6 (60%)
Female	4 (40%)
Age	64–81
Mean age	74
Age range	64–81
Race	
Chinese	9 (90%)
Malay	1 (10%)

The findings were organized into three themes: (1) perceptions of young adults and intergenerational relationships, (2) perceptions of generation gap and navigating intergenerational relationships, and (3) facilitators and barriers of intergenerational relationships and IGPs. In quoted excerpts, B### indicates IGP participants; C### indicates non-IGP participants. Participants primarily communicated in Singlish, a local colloquial variety of English commonly spoken in Singapore, using expressions such as ‘lah’ or ‘hor’, which convey emphasis or tone in casual conversations.

### Theme 1: perception of young adults and intergenerational relationships

This theme examined older adults’ perception of young adults and their relationship with them, specifically exploring the similarities and differences between IGP participants and non-IGP participants.

IGP participants frequently offered warm appraisals of young adults, describing them as respectful, caring, and patient. They appreciated being greeted and helped in everyday situations and noted that young adults were patient in explaining unfamiliar concepts to them, such as learning a new skill. IGP participants also reported that they enjoyed interactions that involved young adults assisting, teaching, or spending time with them.

*“If they enter into a lift and see us, they will greet us with, ‘Aunty’, ‘Aunty, let me help you press’, ‘Aunty, let me carry the things for you… When we were a bit slow to learn things, the young people would say, ‘Ah Ma, do not worry, we will teach you’… made us have a peaceful mind that they will teach us and we did not need to be scared.” (B001)*

IGP participants voiced their enjoyment in forming relationships with young adults through interactive programs offered by community centers or active aging centers (a go-to point, situated within communities, that extends support to older adults). They described a sense of increased ‘liveliness’ when attending IGPs and reported that they looked forward to future visits by young adults.

*"The students came and played games with us…. Now we have a new toy and are very happy. When we gather all together, we’re happy. Young people played the new game with us and we had a good time. [When they] were going to go back, we would miss them and ask when they would come back again. We imagined when they would come back to play with us." (B001)*

However, IGP participants viewed forming deeper relationships with young adults as somewhat unnecessary and preferred to engage in small talk, cheerful banter, or activity-bound interactions. They perceived young adults to be busy and felt a need to consider the appropriate depth of their relationship so as to not take up more of young adults’ time than necessary. One participant articulated this belief:

*"When they come, they bring with them an air of cheerfulness. “Have you eaten? How are you?”...They greet us, “uncle, auntie, how are you?” From about two hours of interaction, the seniors will be very happy…Just like when I’m downstairs, someone greets me “good morning”, that will do. They are busy working or studying, so we have to understand." (B006).*

On the other hand, non-IGP participants expressed neutrality toward young adults and had no strong positive or negative opinions of them. Older adults highlighted that unless there were structured activities or programs with the purpose of bringing young adults and older adults together, the two generations have very few opportunities to interact and will typically lead their lives separately.

*"Maybe if those young people are doing [volunteer work] they will [interact with you]. Those normal young people won't bother to engage with old people. Outside, you get together, it's good. Will they bother to chat with you? They won't." (C020)*

*"Because we're so old. We rarely encounter young ones, don't even talk…[we’re not] close to the young ones." (C021)*

A few older adults reported encountering young adults who were rude or appeared disinterested in them. They observed that young adults were generally more educated than they were, and this difference in education was described as reinforcing perceptions of arrogance among young adults and contributing to social distancing between the generations.

*"...their education is at a much higher level. Their thinking is different, to me, young people lack one thing: consideration." (C016)*

Similarly to IGP participants, non-IGP participants characterized their interactions with young adults as predominantly superficial, typically limited to pleasantries, with little expectations of follow-up or relationship development.

*“Oh, we didn’t talk about the other’s matter, eh, just ask what you do, where do you work, and so on, how are you, ah, uncle have you retired [laughs]. Just some talk.” (C024)*

### Theme 2: perceptions of the generation gap and navigating intergenerational relationships

This theme considered how older adults perceived generational differences, including understanding how differences occurred, and how they navigated intergenerational relationships.

### Theme 2.1: perceptions of generation gap

Both IGP participants and non-IGP participants reported that the differences were a result of typical individual-level differences that occured between individuals regardless of age. They felt that some people, irrespective of generation, might not get along because of different temperaments and traits.

*"No lah. Some [people] are good. Some [people] are no good. We know it." (B013)*

*“…[relationship] depends on personality. Some young people won’t talk to you so much…Some won’t, some are good, and some are kind. It’s the individual personality.” (C006)*

Both groups of older adults also attributed differences between themselves and young adults to individual mindsets. They regarded intergenerational relationships as largely contingent on affinity, emphasizing that compatibility was shaped more by individual rapport than by generational membership.

*"Why there is a difference. I can say maybe different individuals’ mentality... Depending on the individual’s mentality…It’s still an individual’s mentality. Some children are very nice, and young adults are also very nice." (B012)*

*"Everyone has their own way of thinking...it's like they say, it's a matter of life. Maybe if they meet someone that's fated, you get along...I say the one downstairs at the community club...he seems to get along with me, it's strange [laughs]...he's young...it depends on the individual, really." (C020)*

Conversely, some IGP participants reported “no differences” between interacting with young versus older adults. They reported that age was largely irrelevant; ease of interaction depended on the individual and shared topics, and they engaged others in the same way regardless of age.

*"To me, everyone also same lah. No doubt young or what, it’s the same lah. No difference from them lah." (B011)*

### Theme 2.2: navigating intergenerational relationships

IGP participants reported that they were attentive to young adults’ preferences and were concerned with how they might be perceived by them. However, older adults reported that such attentiveness and concerns were rarely extended to peer-to-peer interactions. For instance, many older adults perceived young adults as busy and therefore avoided prolonging interactions with them.

*“They have their own [lives]. They have their things. Do not want to trouble them too much." (B014)*

Some older adults also reported feeling concerned about unintentionally offending young adults and therefore filtered their speech.

*“You can say anything to old people. When you say something to young people, you worry that you might offend them and it is not good." (B002)*

Moreover, other participants shared that they derived enjoyment from seeing young adults enjoy the activities, highlighting their willingness to defer to young adults’ preferences.

*“If I like [activities] and they don’t like [activities] then also no point doing. What’s most important is that the [young adults] like it, we [older adults] will confirm like it one." (B003)*

On the other hand, older adults reported that they were largely unconcerned with how young adults might view them and were uninterested in forming intergenerational relationships. Participants cited a sense of disconnect that reduced their motivation to manage impressions, which many of them attributed to the difficulty in making friends as they aged and young peoples’ lack of initiative to understand them.

*"Make what friends, we're old already, make what friends. No, some people hor, don't want to make friends with you." (C003)*

*"There's nothing to talk about. [Laughs] Like they have their own...only ask things like, have you eaten, how have you been?...They don't try to understand me [laughs]." (C016)*

### Theme 3: facilitators and barriers of intergenerational relationships and IGPs

This theme discussed how both IGP and non-IGP participants perceived the formation of intergenerational relationships and programs, encompassing the barriers and enablers that shaped these interactions.

### Theme 3.1: friendship, skill development, and understanding differences as facilitators of intergenerational relationships

IGP participants described joining programs to lift mood, make friends, and learn from young adults. Sessions were seen as a structured, cheerful space that provided encouragement during difficult periods and opportunities to pick up new skills. One participant noted how attending IGPs helped her cope and feel more positive:

*“At the time, I started to have mild depression…They knew my condition and asked me to think positively and come down to make myself…happy for a while. Then, I learned to take things easy and opened my heart again.” (B001)*

Other participants expressed joy in forming friendships, enjoying the companionship and social interaction with others, including young adults during the IGP programs.

*“Yes, when we play together, I feel happy…we also feel very happy in each other’s company…Once you walk out…there are a lot of friends to talk to already!” (B001)*

Some IGP participants also reported learning new skillsets, such as familiarizing themselves with modern technology, through their interactions with young adults.

*“We are old, there are a lot of things we don’t know. So [I] must learn from the young guy lah… More or less they teach us we have some knowledge lah.” (B011)*

IGP participants emphasized the importance of understanding and adapting, as a means to maintain harmony despite generational differences.

*“Today’s children are not like those in the past, parents are like friends with their children, then families will be happy. If you are strict and overbearing, the child will rebel and do the opposite.” (B003)*

### Theme 3.2: perceived differences, individuality, skepticism, and practical constraints as challenges to intergenerational relationships

Among IGP participants, several older adults described a gap in grasping how young adults thought and, as a result, prevented closer bonds from being formed. Perceived differences in “mentality” and language, among others, were commonly cited as shaping generational outlooks and everyday talk.

*“I think for us seniors, we have difficulties understanding how youths think. So, we do not interact with them too often…They have their own mentalities. Our thinking is ‘take the days as they come’. Different ways of thinking.” (B006)*

Some participants also perceived both generations to be more individualistic and more focused on their lives due to the lack of commonalities, thus resulting in feelings that individuals led separate lives and are engaged in their individual activities and interests.

*"Generally, young adults and older adults...you lead your lives, we lead our lives, this is the phenomenon in Singapore now. Unlike a 70 year old man to become good friends with a 20-year-old, have a common topic to talk about, share the same hobby..." (B014)*

Non-IGP participants voiced similar challenges: topics and interests often did not match, and different life stages brought different priorities, making deeper conversations unlikely even when casual chat was easy.

*“The topic, they don’t quite match. They say this, we say that, it’s no good. They’re just young people. Old people don’t quite [understand]. They don’t quite match, right, like this.” (C007)*

For non-IGP participants, skepticism stemmed from a sense that the scope for ‘bridging’ was limited. They were doubtful that such efforts were either needed or would deliver more than minimal improvement. One participant described their thoughts when asked about intergenerational relationships with younger adults:

*“…friends, meaningful just for…casual chit-chat…but for deeper communication… not so easy…there is always room for improvement, but the improvement is…very slight, 5% to at the very most 10%.” (C019)*

A number of participants also questioned the necessity of attempting to bridge the gap between older adults and young adults, given their perception of fundamentally different lived experiences, motivations, and priorities.

*“Is there a need? You have to see…why do you want to do that? What’s your objective?…Because our lives, [young adults’] lives are different…why do you want to narrow the [generation gap]?” (C016)*

Older adults also highlighted the practical barriers that limited participation. For example, health concerns that restricted mobility and fatigue, made attendance at IGPs difficult.

*“…it’s too much effort, can’t talk [for] too long… Because the doctors [say] I have heart disease.” (C020)*

Another consideration among non-IGP participants was personal preference. For example, discomfort with large crowds, interpersonal friction, or limited interest in the activities offered. Some older adults also preferred to prioritize their own routines when schedules overlapped (e.g., caregiving or household tasks), which reduced their availability for IGPs.

*“…previously, I didn’t have things to do, so I had more free time. [But now] I go over to my son’s house to help… it’s tiring… I told them I won’t go anymore, it’s too tiring. I don’t like it.” (C007)*

## Discussion

This study compared the experiences of older adults who had participated in IGPs, examining their perceptions of young adults, intergenerational relationships and the generation gap, and the facilitators and challenges of intergenerational relationships and IGPs. Three core themes emerged, interpreted through Allport’s Intergroup Contact Theory to highlight the impact of positive intergroup contact, and the SST to explain the challenges in sustaining long-term, meaningful intergenerational relationships within the larger context of an Asian community.

Our findings revealed that older adults’ perceptions of young adults shaped the quality of the interactions with them. Participants who participated in IGPs perceived young adults to be largely approachable and compassionate, reflecting the benefits of positive intergroup contact. They also expressed an awareness of how they were perceived by young adults and were motivated to present themselves positively. In contrast, non-IGP participants held more neutral impressions of young adults and appeared less concerned with creating or maintaining a favorable impression. However, both groups viewed intergenerational relationships as casual: IGP participants enjoyed their interactions with young adults but held few expectations that these relationships would be sustained, whereas non-IGP participants expressed little expectations or desire for friendships with young adults. Our findings corroborated Pettigrew and Troop’s meta-analysis of 515 studies, which found that intergroup contact, regardless of whether the four conditions were met (equal status, cooperation, shared goal and institutional support) significantly improved outgroup attitudes (9, 10). However, these positive perceptions did not necessarily translate into expectations of sustained relationships, as both groups of older adults showed preference for same-age friendships over intergenerational friendships.

Older adults’ reflection on the generation gap revealed nuanced understanding of the differences and the effort required to navigate intergenerational relationships. Most participants attributed the perceived gap to differences in interests, mindset, and personality. This was consistent with research demonstrating that, even across diverse cultures, age-related stereotypes and attitudes persisted, indicating how societal norms and cultural narratives can essentialize personality differences into generational categories (27). Our results similarly reflected the manner in which participants described their perceptions of the generation gap; however, their attitudes toward whether the gap could be bridged varied. Participants who did not participate in IGPs viewed the generation gap as fixed and unbridgeable, often positioning differences in personality and mindset as insurmountable. In contrast, IGP participants believed that the gap can be bridged, likely drawing on previous positive experiences with young adults during IGPs, as evidence that such differences can be negotiated. Nonetheless, IGP participants described being cautious in their interactions, such as being mindful to not offend young adults, reluctant to take up their time, and sometimes deferring to young adults’ preference for activities. These differing perspectives suggested that direct contact fostered openness to bridge the generation gap, whereas an absence of this contact perpetuated the notion that the gap is permanent (28, 29). Unlike same-age friendships, where individuals in similar life stages share experiences, cultural references, and communication styles that facilitate mutual understanding (30, 31), intergenerational relationships often require bridging differences in values, norms, and communications, demanding greater effort to sustain them (31).

Moreover, these behaviors reflected cultural norms of respect and deference often observed in Asian context, such as in Singapore. In these setting, intergenerational dynamics may be shaped by cultural expectations of filial piety and respect toward elders, framing intergenerational interactions as hierarchical rather than equal (32). Such dynamics may result in both generations to be cautious of their behaviors: young adults may restrain themselves in interactions with older adults to avoid “overstepping,” while older adults may limit themselves to avoid burdening young adults, resulting in respectful and polite but superficial interactions. Although positive intergroup contact may reduce stereotypes and anxieties through repeated exposure, opportunities for collaboration, shared goals, and participation in tasks that promote equal status among young adults and older adults (33), it may only result in surface-level connections rather than deeper, long-term relationships. Thus, while IGPs create opportunities for contact and the development of intergenerational relationships, its impact may be limited by cultural expectations for friendship.

Our findings identified both facilitators and challenges that influenced their participation in intergenerational relationships and programs. Consistent with Allport’s Intergroup Contact Theory, participation in IGPs fostered positive impressions and greater openness to bridging generation differences. This was evident among IGP participants who highlighted the emotional and social benefits they received from young adults. On the other hand, older adults who did not participate in IGPs cited barriers such as health limitations and competing priorities. Notably, some participants questioned the value of bridging the generation gap. Participants who participated in IGPs held mixed views: for some, addressing the generation gap was perceived as possible and worthwhile, but for others, it was viewed as an acceptable and natural difference that did not require bridging. Participants who did not participate in IGPs generally held the latter perspective. While extensive research has demonstrated the value of intergroup contact in reducing stereotypes and fostering positive intergroup impressions, recent literature suggested that contact alone may be insufficient in developing positive relationships between groups (34). Drury et al. found that the quality of the contact was an important predictor of positive age-related attitudes (35). MacInnis and Page-Goulds’ proposed the concept of a contact threshold, the point in which intergroup interactions shift from positive to negative, suggesting that this threshold is achieved when both quantity and quality contact is optimized (16). Therefore, the absence of intergroup contact likely explained why older adults who did not participate in IGPs saw little value in bridging the generation gap. For older adults who did participate, the limited or uneven quality of contact may account for the mixed perceptions toward bridging the generation gap.

Building on this, the perceptions of intergenerational relationships and the generation gap provided further understanding of participants’ perceptions of IGPs. Participants who participated in IGPs generally viewed IGPs positively and were satisfied with casual, surface-level interactions, whereas participants who did not participate in IGPs questioned the value and relevance of IGPs. The SST provides a useful lens to interpret these attitudes: older adults who become increasingly aware of their limited time may de-prioritize IGPs if they are not viewed as being aligned with their emotional goals and satisfaction (36, 37). This was reflected among older adults who participated in IGPs, as they enjoyed interactions with young adults even when they remained surface-level, and in older adults who did not participate, who expressed a preference to avoid IGPs. With age, older adults prioritize creating a network of meaningful and familiar relationships over novel and effortful ones (23, 38). Nikitin et al. found that age was positively associated with perceived meaningfulness of daily social interactions, and that both factors were positively associated with subjective well-being (39). Similarly, Parker and Strough found that although older adults reported smaller social networks, their well-being was more strongly associated with social satisfaction than the reported number of close friends, suggesting that the perceived quality of relationships, rather than quantity, was a stronger predictor of well-being (40). Taken together, these findings may suggest that while IGPs can foster positive impressions of young adults, many older adults may not see them as essential to developing close friendships and their well-being. This may explain why older adults who participated in IGPs, despite having positive experiences with young adults, were content with casual interactions, while non-IGP participating older adults were skeptical about the efficacy of attending IGPs. Thus, the long-term impact of IGPs is shaped not only by opportunities for contact but also by older adults’ motivation and social goals.

This is aligned with SST, which suggests that older adults’ prioritization of meaningful and familiar goals may limit the extent to which these interactions develop into deeper relationships. Extensive research has demonstrated the value of intergenerational relationships in promoting social cohesion, mutual understanding, and well-being (41–43), however the persistence of perceived generation gaps and ambivalence of whether such gaps are worth bridging further underscore that contact alone is insufficient. Rather, what might be needed is a mindset shift to view intergenerational relationships as possible, valuable, and emotionally rewarding. The effectiveness of IGPs depends not only on creating opportunities for positive contact but also on aligning these opportunities with older adults’ goals, expectations, and readiness to invest in intergenerational relationships.

### Implications

The findings offer several insights into the development and implementation of IGPs.

### Structuring programs that align with older adults’ goals and interests

Our findings demonstrated that older adults often enter IGPs with specific personal goals or may not see the value of participating if the activities do not align with their priorities. This underscores the need to structure programs that genuinely align with older adults’ interests and goals. To remain meaningful, programs need to recognize and cater to the diverse needs and interests of older adults, rather than assuming a homogeneous set of motivations or focusing solely on task completion. This may include having both young adults and older adults co-create IGPs that reflect the goals and interests of both generations.

### Creating emotionally meaningful contact

Programs should focus on creating emotionally meaningful and engaging interactions that align with older adults’ goals, as opposed to simply increasing the number of intergenerational contacts. For instance, having trained facilitators can help set the tone of IGPs by encouraging participants to approach the program with respect, reciprocity, and curiosity. Moreover, they play a crucial role in nurturing deeper conversations while creating a psychologically safe environment for participants to share stories, ask questions, and better understand each other’s perspectives. By prioritizing the quality of interactions over quantity, programs can better foster genuine connection, mutual learning, and sustained engagement.

### Ensuring flexibility and accessibility for older adults

Programs that offer activities that are not overly demanding, complex, or rigid are more likely to attract and retain participation from older adults. Programs can be structured to be more cognitively and physically inclusive. For instance, they should avoid overly complex tasks, use clear and simple instructions, and present information in short and manageable segments that older adults can navigate independently. Moreover, programs need to account for variations in hearing, vision, stamina, and mobility by offering amplified audio, providing written instructions in larger fonts, ensuring comfortable seating, and incorporating breaks or shorter activities. Activities should also offer alternatives so that older adults with differing levels of function can participate meaningfully. By integrating these considerations, programs become more welcoming, inclusive, and empowering for older adults.

### Strengths and limitations

There are several strengths to this study. The study examined and compared the experiences and perceptions of participants and non-participants of IGPs, providing a more nuanced understanding of the factors influencing engagement, priorities and perceived barriers. Additionally, the study integrated frameworks, such as Allport’s Intergroup Contact Theory and SST, to interpret findings, offering a comprehensive lens to understand the complex dynamics of intergenerational interactions. Nevertheless, limitations remain. The inclusion criteria comprised participants who have participated in an IGP within the last 3 years, making participants susceptible to recall bias. In addition, while an interpretive approach yielded broad insights, not all topics were examined in depth. For instance, the nature of IGPs could be further explored to provide more nuance into the types of IGPs that older adults prefer or are less inclined to participate in. Additionally, the authors did not collect data on the nature of IGPs (i.e., type of IGP activities, facilitation structure, intensity and type of IGPs, etc.) which could have contextualized participants’ experiences more comprehensively. Finally, the authors did not have more granular demographic information beyond age, sex, and ethnicity.

### Future directions

Future research could build on these findings. Firstly, longitudinal studies could examine how perceptions of intergenerational relationships in IGPs evolve over time, including interventions that address negative perceptions and barriers. Secondly, studies exploring the specific characteristics of IGPs (e.g., program format, types of activities, duration, etc.) can be conducted to determine program elements that would optimize intergenerational relationships.

## Conclusion

In conclusion, the study highlighted how older adults’ experiences with intergenerational relationships and programs are shaped by both contact and motivation. Allport’s Intergroup Contact Theory explained how participation in IGPs fostered positive impressions of young adults, while the SST explained why older adults may prefer casual interactions over deeper intergenerational relationships. The persistence of the perception of the generational gap suggests that contact alone is insufficient, and a mindset shift is pertinent in viewing intergenerational relationships and programs as rewarding and meaningful.

## Data Availability

The raw data supporting the conclusions of this article will be made available by the authors, without undue reservation.
